# Interfacial B-Site Ion Diffusion in All-Inorganic Core/Shell Perovskite Nanocrystals

**DOI:** 10.1021/acsnano.3c05876

**Published:** 2023-11-14

**Authors:** Shuya Li, Hanjie Lin, Chun Chu, Chandler Martin, Walker MacSwain, Robert W. Meulenberg, John M. Franck, Arindam Chakraborty, Weiwei Zheng

**Affiliations:** † Department of Chemistry, Syracuse University, Syracuse, New York 13244, United States; ‡ Department of Physics, Syracuse University, Syracuse, New York 13244, United States; § Department of Physics and Astronomy and Frontier Institute for Research in Sensor Technologies, University of Maine, Orono, Maine 04469, United States

**Keywords:** core/shell nanocrystals, lead-free perovskites, ion diffusion, optical properties, enhanced stability

## Abstract

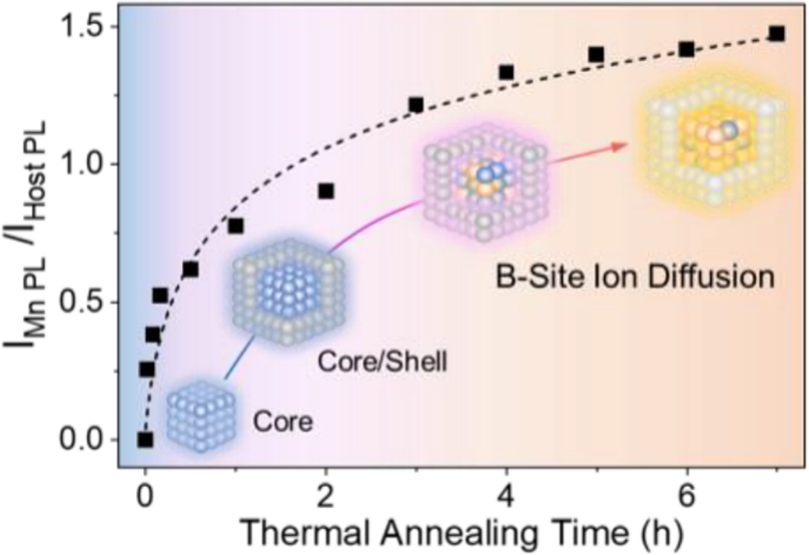

All-inorganic metal halide perovskites (ABX_3_, X = Cl, Br, or I) show great potential for the fabrication of optoelectronic devices, but the toxicity and instability of lead-based perovskites limit their applications. Shell passivation with a more stable lead-free perovskite is a promising strategy to isolate unstable components from the environment as well as a feasible way to tune the optical properties. However, it is challenging to grow core/shell perovskite nanocrystals (NCs) due to the soft ionic nature of the perovskite lattice. In this work, we developed a facile method to grow a lead-free CsMnCl_3_ shell on the surface of CsPbCl_3_ NCs to form CsPbCl_3_/CsMnCl_3_ core/shell NCs with enhanced environmental stability and improved photoluminescence (PL) quantum yields (QYs). More importantly, the resulting core/shell perovskite NCs have color-tunable PL due to B-site ion diffusion at the interface of the core/shell NCs. Specifically, B-site Mn diffusion from the CsMnCl_3_ shell to the CsPbCl_3_ core leads to a Mn-doped CsPbCl_3_ core (i.e., Mn:CsPbCl_3_), which can turn on the Mn PL at around 600 nm. The ratio of Mn PL and host CsPbCl_3_ PL is highly tunable as a function of the thermal annealing time of the CsPbCl_3_/CsMnCl_3_ core/shell NCs. While the halide anion exchange for all-inorganic metal halide perovskites has been well-developed for band-gap-engineered materials, interfacial B-site diffusion in core/shell perovskite NCs is a promising approach for both tunable optical properties and enhanced environmental stability.

## Introduction

All-inorganic metal halide perovskites (ABX_3_, X = Cl, Br, or I) have excellent light absorption, tunable band gap, and high charge carrier mobilities, which offer great potential for optoelectronic devices, such as photovoltaics,^1,2^ electrochemical sensors,^3,4^ light-emitting diodes (LEDs),^5,6^ and photocatalysis.^7,8^ However, the toxicity and instability of lead-based perovskites, such as CsPbX_3_, limit their applications.^9,10^ To address these issues, lead-free halide perovskite materials have recently attracted attention due to their lower toxicity and higher stability as alternatives to lead-based perovskite nanocrystals (NCs).^6,11−13^ Some low-toxicity constituents with a perovskite structure include Sn/Ge-based halides,^14,15^ double perovskites,^16,17^ and Bi/Sb-based halides.^18,19^ Mn^2+^ ions are also considered potential B-site ions to fabricate perovskite-type materials (e.g., CsMnCl_3_) for the application of X-ray imaging and LED devices^20,21^ and can also serve as dopants to significantly tailor the optical properties.^7,22−24^ Though ideal lead-free candidates could have low toxicity, tunable direct band gaps, high optical absorption coefficients, and compatible stability, the performances of lead-free perovskites are still not yet approaching the spectacular performance of lead-based perovskites (APbX_3_).^12^ Therefore, finding a method to effectively utilize the merits of lead-based and lead-free perovskite materials is essential for wide applications of perovskites.

Shell passivation for core/shell structured NCs is one of the promising strategies to improve the environmental stability of perovskites and to tailor the optical properties by removing the surface defects.^25−28^ It is highly desirable to obtain core/shell lead-based and lead-free perovskite NCs to achieve the maximum utilization of perovskite materials. Core/shell FAPbBr_3_/CsPbBr_3_ (FA = formamidinium) organic–inorganic hybrid perovskite NCs with the same B-site and halide ions were reported to have high efficiency and improved stability for LED applications.^26^ However, so far, there have been very limited reports on the epitaxial growth of all-inorganic perovskite shells on the surface of perovskite NCs. The limited report of all-inorganic core/shell perovskite NCs reflects the challenges of epitaxial shell growth for perovskites compared to traditional II–VI (e.g., CdS and CdSe), III–V (e.g., GaAs), and IV–VI (e.g., PbSe and GeTe) semiconductor NCs,^29−31^ due to the soft ionic nature and fast anion exchange of lead halide perovskites.^32^

**Figure 1:**
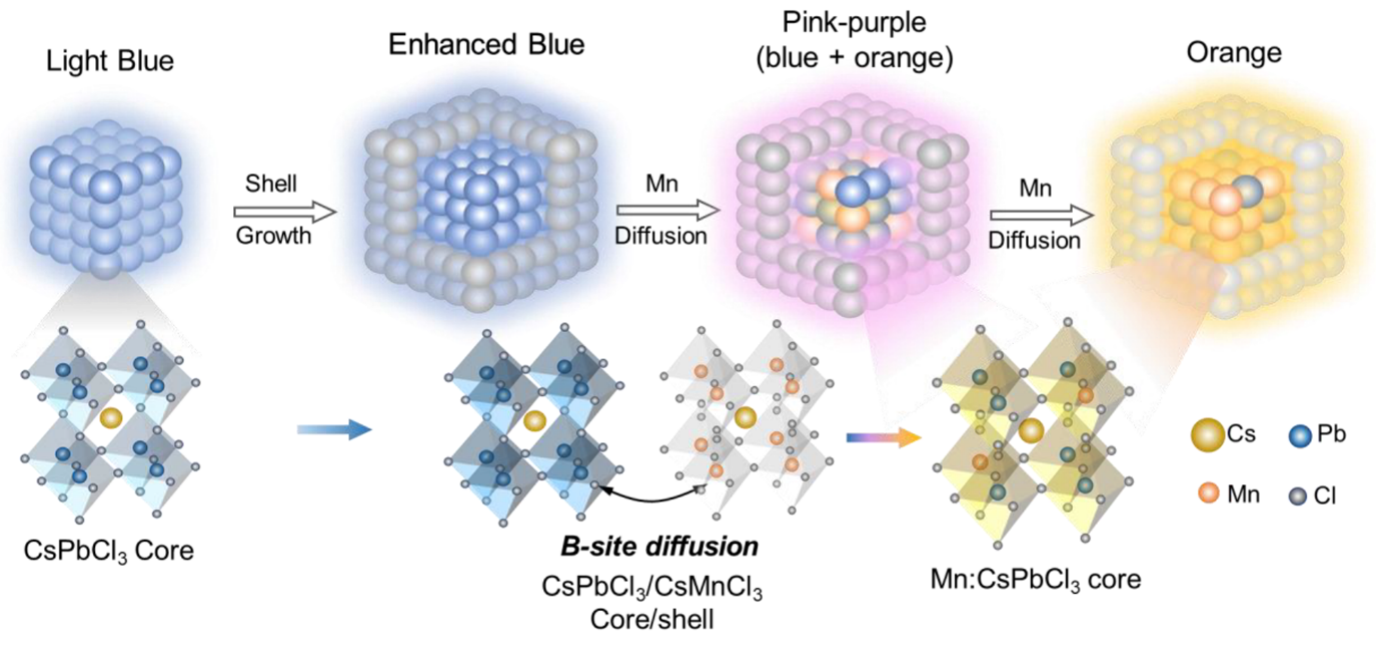
B-site ion diffusion at the interface of the CsPbCl_3_/CsMnCl_3_ core/shell structure to efficiently tune their optical properties, improve the environmental stability, and reduce the toxicity of lead-based perovskite materials.

Herein, we developed a two-pot synthesis process for the epitaxial growth of the lead-free CsMnCl_3_ shell on the surface of CsPbCl_3_ to obtain CsPbCl_3_/CsMnCl_3_ core/shell NCs. The wide band gap of the CsMnCl_3_ shell leads to a type I core/shell structure, which can improve the photoluminescence (PL) quantum yields (QYs) of the blue light emission from the CsPbCl_3_ core NCs. Moreover, the environmentally friendly CsMnCl_3_ shell serves as a protective layer that can prevent the core from degrading under moisture, heat, and light irradiation. Interestingly, interfacial B-site ion diffusion and exchange of the CsPbCl_3_/CsMnCl_3_ core/shell NCs was observed, resulting in the formation of Mn-doped CsPbCl_3_ (i.e., Mn:CsPbCl_3_) core (Figure 1), which leads to a highly tunable ratio of the Mn^2+^ PL (orange light) and host PL (blue light) in the visible range controlled by varying the thermal annealing time. At the early stage of thermal annealing with a small amount of Mn^2+^ ions being diffused into the CsPbCl_3_ core, the core/shell perovskite NCs emit a pink-purple color, resulting from the combination of the blue and orange light. However, extended thermal annealing (∼5–7 h) with high Mn^2+^ doping concentration inside the CsPbCl_3_ core from the B-site ion diffusion leads to an orange color emission. Instead of tuning the photophysical properties by altering the halide ions in traditional designs of all-inorganic metal halide perovskites NCs,^33−35^ the interfacial ion diffusion in perovskite core/shell NCs provides a feasible method to tailor local perovskite composites, improve PL QYs, and enhance environmental compatibility.

## Results and Discussion

**Figure 2:**
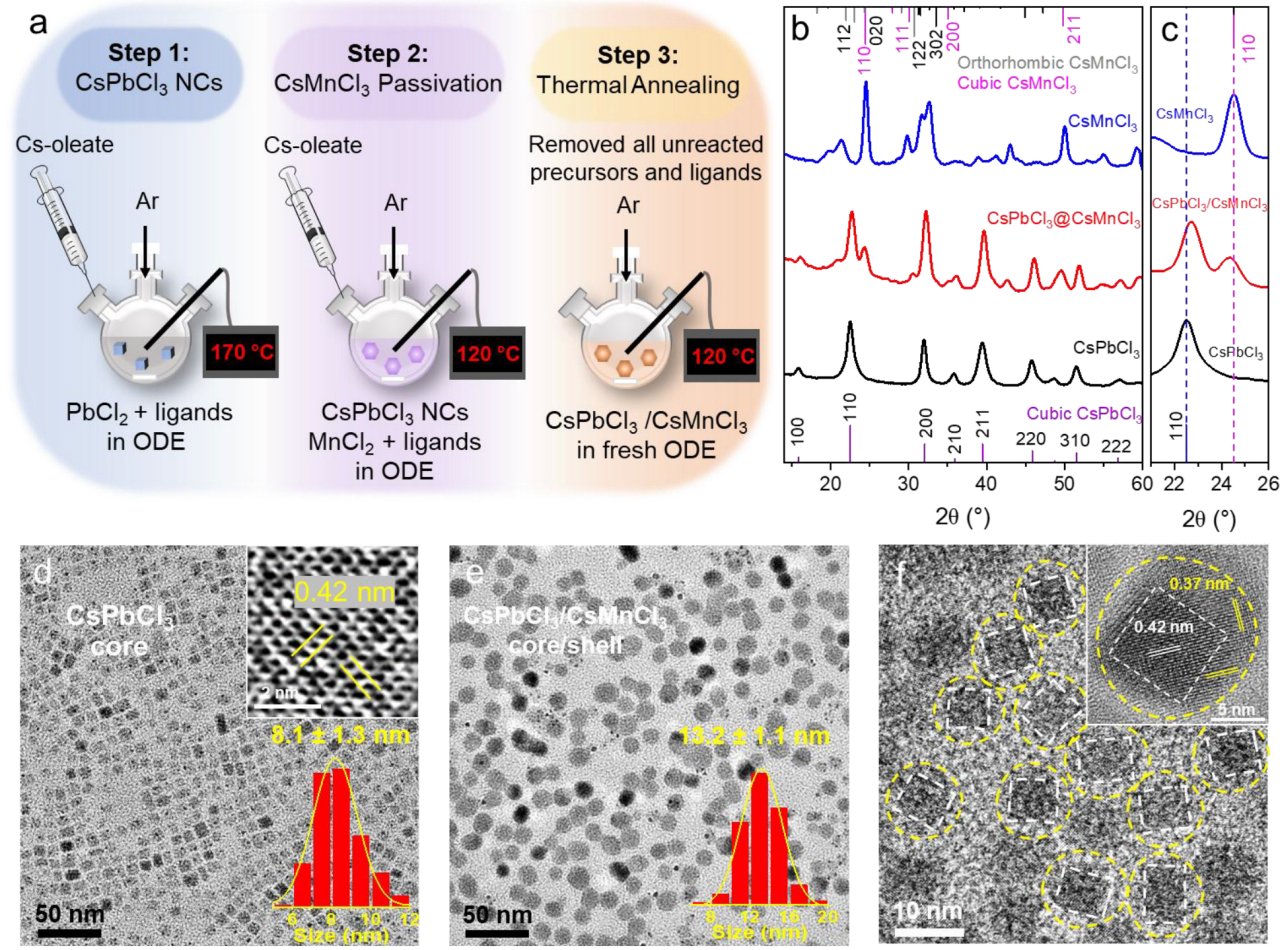
(a) Schematic illustration of the growth of the CsPbCl_3_ core and the CsPbCl_3_/CsMnCl_3_ core/shell NCs, followed by thermal annealing at 120 °C up to 7 h. (b) XRD patterns for CsPbCl_3_ NCs (black), CsPbCl_3_/CsMnCl_3_ core/shell NCs (red, thermally annealed at 120 °C for 7 h), and pure CsMnCl_3_ (blue). (c) Zoom-in XRD patterns showing peak shifting of the (110) diffraction peaks of CsPbCl_3_ and CsMnCl_3_ in the CsPbCl_3_/CsMnCl_3_ core/shell NCs. (d) TEM image of the CsPbCl_3_ core. Insets are the high-resolution TEM image showing *d* spacing of the (110) lattice plane and the histogram of particle size. (e) TEM image of CsPbCl_3_/CsMnCl_3_ core/shell NCs. The inset is the histogram of particle size. (f) Zoom-in TEM image showing the frames of the CsPbCl_3_ core (white) and the CsMnCl_3_ shell (yellow) of the CsPbCl_3_/CsMnCl_3_ core/shell NCs. Inset: high-resolution TEM image showing the lattice of (110) planes of the CsPbCl_3_ core NCs and CsMnCl_3_ shell NCs with *d* spacings of 0.42 and 0.37 nm, respectively.

In this work, CsPbCl_3_/CsMnCl_3_ core/shell NCs were developed for enhanced environmental stability and optical properties. We first synthesized CsPbCl_3_ NCs by using a hot injection method (Step 1 in Figure 2a).^33^ To enhance the stability and minimize the environmental impact of the Pb-based perovskite NCs, shell passivation with a Pd-free CsMnCl_3_ shell was performed for CsPbCl_3_/CsMnCl_3_ core/shell NCs. The as-synthesized CsPbCl_3_ NCs were added to a mixture of MnCl_2_ and ligands (i.e., oleic acid (OA) and oleylamine (OAm)) in 1-octadecene (ODE) (Step 2 in Figure 2a). Then, the gradual addition of the Cs-oleate precursor at 120 °C allowed the epitaxial growth of cubic CsMnCl_3_ shell NCs on the surface of cubic CsPbCl_3_ core NCs, due to the same crystal structure and similar lattice parameters. For example, the *d* spacings of CsPbCl_3_ (110) and CsMnCl_3_ (110) planes are 0.396 and 0.362 nm, respectively, with lattice mismatch of less than 9%. The structural and optical properties of the core/shell NCs were further studied by thermal annealing up to 7 h (Step 3 in Figure 2a).

The XRD pattern of the CsPbCl_3_ core is consistent with that of cubic phase CsPbCl_3_ NCs (black in Figure 2b). Pure CsMnCl_3_ NCs were also synthesized using a hot-injection method to obtain the crystal structure of CsMnCl_3_ (see details in Experimental Section), showing a mixture of cubic and orthorhombic crystal phases of CsMnCl_3_ (blue in Figure 2b).^20,36^ It is likely that the cubic phase of CsMnCl_3_ first epitaxially grows on the surface of CsPbCl_3_ core NCs with the same cubic structure, and a phase transition could occur during thermal annealing,^37,38^ where the cubic phase of CsMnCl_3_ distorted to a pseudocubic phase and then further transferred to the orthorhombic phase. In addition, the orthorhombic structure is closely inter-related with the cubic structure with 90° between all three axes but with slightly different lattice parameters along the *x*, *y*, and *z* directions.^39^ Considering the soft ionic bonding of the perovskite lattice, the lattice distortion at the core/shell interface might also allow possible epitaxial growth of orthorhombic CsMnCl_3_ directly on the surface of cubic CsPbCl_3_ NCs. The CsPbCl_3_/CsMnCl_3_ core/shell NCs display an XRD pattern of the mixture of CsPbCl_3_ and CsMnCl_3_ (red in Figure 2b). Interestingly, in the mixture of CsPbCl_3_ and CsMnCl_3_ diffraction patterns, both CsPbCl_3_ (110) and CsMnCl_3_ (110) peaks from CsPbCl_3_ and CsMnCl_3_ slightly shifted toward each other in the core/shell NCs compared to the pure core and shell NCs (Figure 2c). Specifically, the CsPbCl_3_ (110) peak shifts from 22.52 to 22.72° and the CsMnCl_3_ (110) peak shifts from 24.50 to 24.26°, which indicates the change of the lattice parameters due to the ion diffusion between B-site ions (i.e., Mn^2+^ and Pb^2+^) considering the same A-site ions and X-site halides of the core and shell lattice. The 0.2° increment of the CsPbCl_3_ (110) peak is due to the substitution of larger Pb^2+^ ions (133 pm) by small Mn^2+^ ions (97 pm) via B-site ion diffusion at the interface of the core/shell NCs. Oppositely, the 0.24° decrement of the CsMnCl_3_ (110) peak is consistent with lattice expansion with the incorporation of the larger Pb^2+^ ions in place of Mn^2+^.

Transmission electron microscopy (TEM) image of the core CsPbCl_3_ NCs (Figure 2d) indicate CsPbCl_3_ nanocubes with 8.1 ± 1.3 nm edge dimension, and a *d* spacing of 0.42 nm from the (110) crystal planes (inset of Figure 2d).^40^ The CsPbCl_3_/CsMnCl_3_ core/shell NCs show a sphere-shaped morphology that is similar to the morphology of CsMnCl_3_ NCs (Figure S1)^20^ with a bigger particle size of 13.2 ± 1.1 nm (Figure 2e). Such a morphology change from nanocubes to nanospheres is ascribed to the epitaxial growth of sphere-shaped cubic CsMnCl_3_^36^ on the surface of cubic CsPbCl_3_. A higher magnification TEM image shows the core and shell parts, highlighted with white and yellow frames, respectively (Figure 2f). The high-resolution TEM (HR-TEM) image gives a *d* spacing of 0.42 nm from the (110) plane in the CsPbCl_3_ core (∼8.0 nm in diameter) and a *d* spacing of 0.37 nm for the (110) plane of the outward CsMnCl_3_ shell (inset of Figure 2f), which is in great consistency with the HR-TEM data obtained from the CsMnCl_3_ NCs (inset of Figure S1).^20^ These results suggest that the shell successfully grew on the surface of the core without affecting the original morphology and crystal structure of the CsPbCl_3_ core NCs.

**Figure 3:**
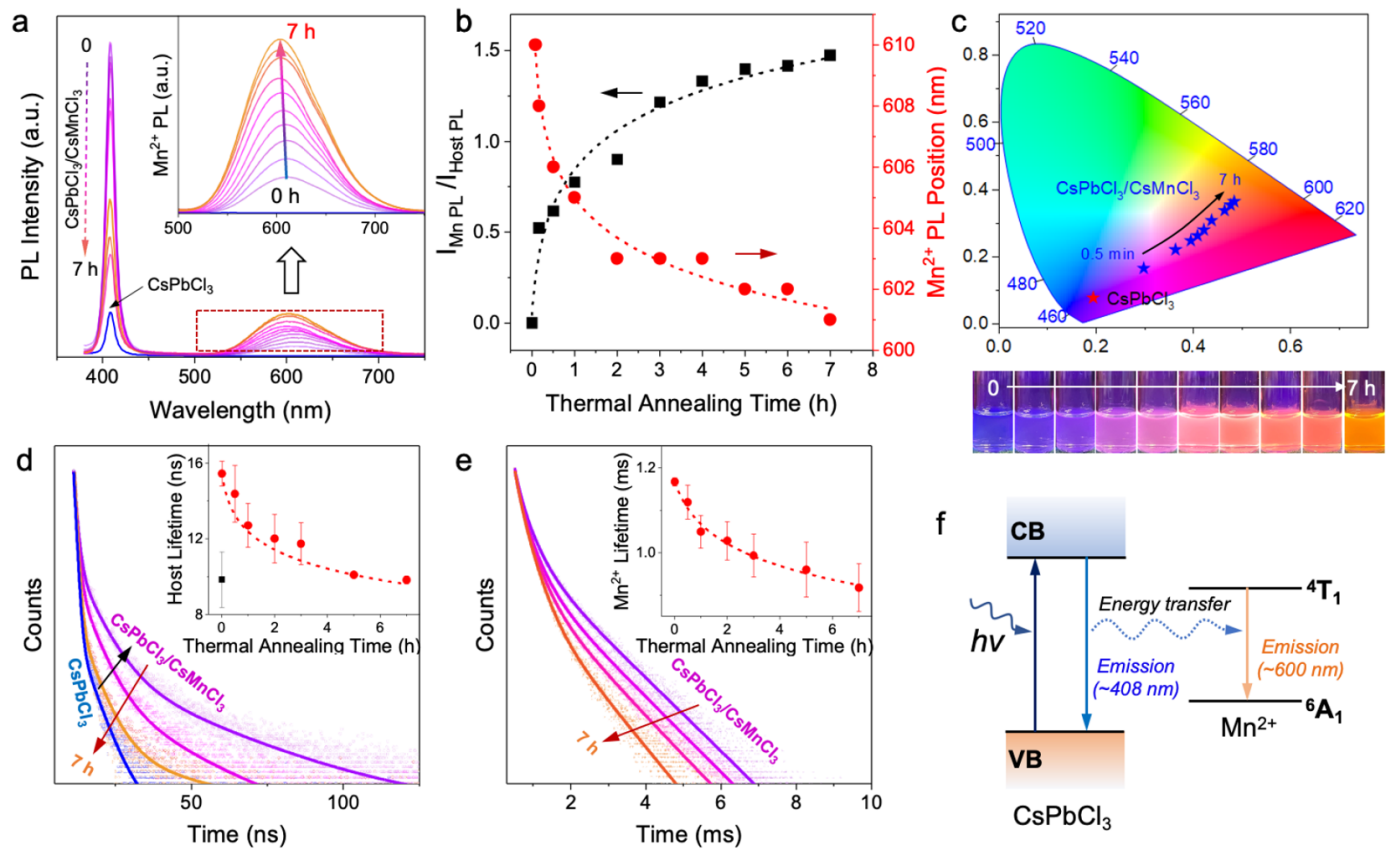
(a) PL spectra of the CsPbCl_3_ core and CsPbCl_3_/CsMnCl_3_ core/shell NCs with different thermal annealing times. Inset: the zoomed-in Mn^2+^ PL over time. (b) Ratio between Mn^2+^ PL and host PL (black squares) and the corresponding Mn^2+^ PL positions (red dots) over thermal annealing time. (c) Chromaticity coordinates (top) and optical images under UV light (bottom) of CsPbCl_3_ NCs and CsPbCl_3_/CsMnCl_3_ core/shell NCs with different thermal annealing times. (d) Host PL lifetime and (e) Mn^2+^ PL lifetime (insets: average PL lifetime) for the samples with respect to thermal annealing time. Errors were calculated as the standard deviation of a population of three repeated samples for each sample. (f) Schematic illustration of the band alignment of the Mn:CsPbCl_3_ NCs and the Mn PL from host-to-dopant energy transfer.

Since the ion diffusion in the solid lattice is generally slow and time-dependent, the photophysical properties of the core/shell NCs with various thermal annealing times were then examined to reveal the B-site diffusion process (Step 3 in Figure 2a). The PL spectrum of the CsPbCl_3_ core NCs displays a blue emission at 408 nm with a PL QY of 6.1% (blue in Figure 3a). As the shell grows, a new Mn PL peak rises at 605 nm from the Mn^2+^ ions, which is consistent with Mn ^4^T_1_ → ^6^A_1_ transition inside the perovskite as well as metal chloride systems.^7,22−24^ It should be noted that the Mn PL position is sensitive to the crystal field splitting, coordination environment, and dopant location.^41−44^ Therefore, such a change in PL spectra after CsMnCl_3_ shelling could indicate the Mn^2+^ diffusion from the CsMnCl_3_ shell to the CsPbCl_3_ core, resulting in the formation of Mn-doped CsPbCl_3_ NCs (i.e., Mn:CsPbCl_3_). In the Mn-doped core NCs, the host CsPbCl_3_ NCs absorb visible light to form excited electrons in the conduction band (CB). In the presence of Mn^2+^ dopant ions, the energy transfer from the CB of the host NCs to Mn^2+^ and the sequential Mn PL from the ^4^T_1_ to ^6^A_1_ transition can occur (Figure 3f), rendering dual-band emission at ∼408 nm (blue color) and ∼605 nm (orange color).

The wide band gap of the CsMnCl_3_ shell has resulted in a type I CsPbCl_3_/CsMnCl_3_ core/shell structure, which improved the total PL QY to ∼28.5%. As shown in the inset of Figure 3a, the Mn^2+^ PL intensity continually increases during the thermal annealing process from 0 to 7 h. The ratio between the Mn^2+^ PL and host PL over the thermal annealing time (black squares in Figure 3b) shows a significant increase of the Mn^2+^ PL contribution from 0 in the CsPbCl_3_ core NCs to 1.48 in the core/shell NCs after B-site ion diffusing for 7 h. With varied ratios of Mn PL and host PL, the emission color of the core/shell NCs was continuously tuned from a nearly pure blue light to purple-pinkish light and finally stabilized with an orange light emission after being thermally annealed for 7 h as indicated by the Commission International de l’Eclariage (CIE) chromaticity coordinates (i.e., from (0.19, 0.09) to (0.48, 0.39)) and the optical pictures under UV light irradiation (Figure 3c).

In addition, a continuous blue shift of the Mn^2+^ PL position is observed with increasing thermal annealing times (a total 9 nm shift, red dots in Figure 3b). One possible explanation for the blue shift of the Mn PL peak is that the Mn^2+^ ions are experiencing different pressures caused by the reduced pure CsMnCl_3_ shell thickness after B-site ion exchange. The shell-thickness-dependent pressure applied onto the Mn dopants in the core NCs based on the spherical symmetric continuum elastic model has been reported, with higher pressure at thicker shells, leading to a Mn PL red shift, and *vice versa*.^45,46^ After Pb^2+^ and Mn^2+^ ion exchange within CsPbCl_3_/CsMnCl_3_ core/shell NCs, reduced pure CsMnCl_3_ shell thickness is expected (i.e., Mn_*x*_:CsPb_1–*x*_Cl_3_/(1 – *x*)CsMnCl_3_) on top of Mn dopant ions in the CsPbCl_3_ core. The reduced pressure on Mn dopants from the thinner CsMnCl_3_ shell could lead to the blue shift of the Mn PL peaks. Moreover, the core/shell NCs showed a significant enhancement for the PL QY compared to the pure CsPbCl_3_ core NCs, and no obvious change of the total PL QY of the core/shell NCs was observed during thermal annealing for 7 h (Figure S2). Therefore, the change of the PL ratio of Mn and host can support the energy transfer from the CsPbCl_3_ host to Mn dopants as Mn ions inwardly diffused into the CsPbCl_3_ core NCs.

Time-resolved PL spectra of the CsPbCl_3_ core and CsPbCl_3_/CsMnCl_3_ core/shell NCs are shown in Figure 3d,e. The average host PL lifetime first significantly increases from 9.8 to 15.5 ns as the shell initially grew on the surface due to the increased PL QY of the type I CsPbCl_3_/CsMnCl_3_ core/shell structure. However, the sequential B-site ion (Mn^2+^ and Pb^2+^) diffusion between the core and the shell leads to a decreased host PL lifetime from 15.5 to 10 ns with increasing annealing time from 0.5 min to 7 h, respectively (inset of Figure 3d), which could be understood as the enhanced host-to-dopant energy transfer at higher Mn doping concentration in the CsPbCl_3_ core NCs (Figure 3f). The Mn^2+^ lifetime of the core/shell NCs decreases with annealing time from 1.19 to 0.92 ms with increasing thermal annealing time from 0.5 min to 7 h (Figure 3e), indicating dopant concentration quenching effects from the increased Mn–Mn short-range interactions at a higher Mn^2+^ doping concentration in the core NCs.

**Figure 4:**
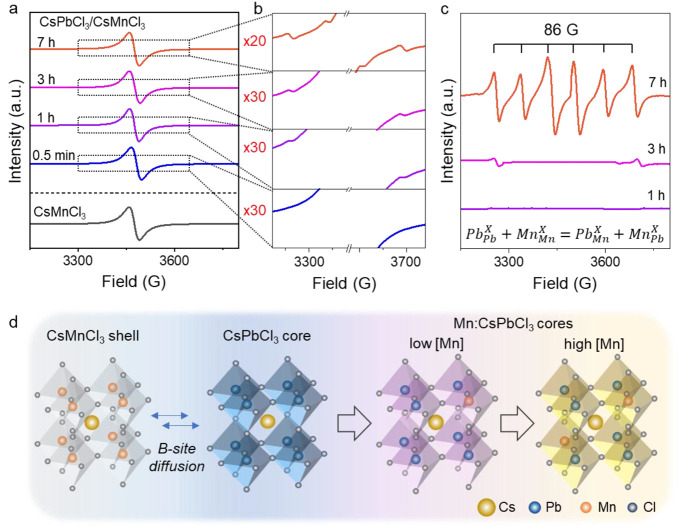
(a) Room-temperature X-band EPR spectra of CsMnCl_3_ NCs and CsPbCl_3_/CsMnCl_3_ core/shell NCs with different thermal annealing times (0.5 min, 1, 3, and 7 h). (b) Zoomed-in and (c) CsMnCl_3_-subtracted EPR spectra showing up to six individual hyperfine splitting peaks of Mn^2+^ dopant ions in the core as the result of ion diffusion after thermal annealing. (d) Schematic illustration of the B-site ion diffusion at the interface of the CsMnCl_3_ shell and CsPbCl_3_ core and the formation of Mn:CsPbCl_3_ cores with a low concentration of Mn dopants at the early stage (purple) and high concentration of Mn dopants at the later stage of the thermal annealing (orange).

Electron paramagnetic resonance (EPR) analysis was also performed to track the change of the chemical environments of Mn^2+^ ions during the B-site ion diffusion process in the core/shell NCs. Upon shelling with CsMnCl_3_ perovskite, CsPbCl_3_/CsMnCl_3_ core/shell NCs show a single broad EPR signal, due to the short-range Mn–Mn interactions in the pure CsMnCl_3_ shell (Figure 4a). The EPR spectrum of the control sample of pure CsMnCl_3_ NCs also only shows a broad EPR peak without hyperfine splitting (black in Figure 4a). Interestingly, Mn hyperfine splitting peaks from isolated Mn^2+^ sites were displayed as the thermal annealing time increased, which is consistent with the change of chemical environment of the Mn^2+^ ions from the pure CsMnCl_3_ phase in the shell to diluted Mn-doped CsPbCl_3_ in the core of the core/shell NCs, as displayed in the zoomed-in EPR spectra (Figure 4b). The B-site ion substitution can be described by using eq 1.
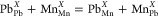
1With the CsMnCl_3_ EPR signal being subtracted, the hyperfine splitting Mn^2+^ signals of the thermally annealed NCs were clearly displayed, as the intensity increased as a function of the thermal annealing time with six intense hyperfine splitting peaks from Mn^2+^ ions for 7 h thermally annealed core/shell NCs (Figure 4c). The hyperfine splitting constant of 86 G indicates that the Mn^2+^ ions were incorporated into the core lattice sites of NCs,^34^ which can further confirm the Mn ion diffusion into the core of the CsPbCl_3_/CsMnCl_3_ core/shell NCs. Figure 4d is a schematic illustration of interfacial B-site ion diffusion at the interface of CsPbCl_3_/CsMnCl_3_ core/shell NCs and the formation of Mn:CsPbCl_3_ core NCs. At the early stage of ion diffusion with a low concentration of Mn^2+^ dopants in the core, the core/shell perovskite emits a pink-purple color resulting from the combination of blue and orange light. At a later stage of B-site ion diffusion with extended thermal annealing time, a high Mn^2+^ doping concentration inside the CsPbCl_3_ core could be obtained; the overall emission turns to an orange color.

**Figure 5:**
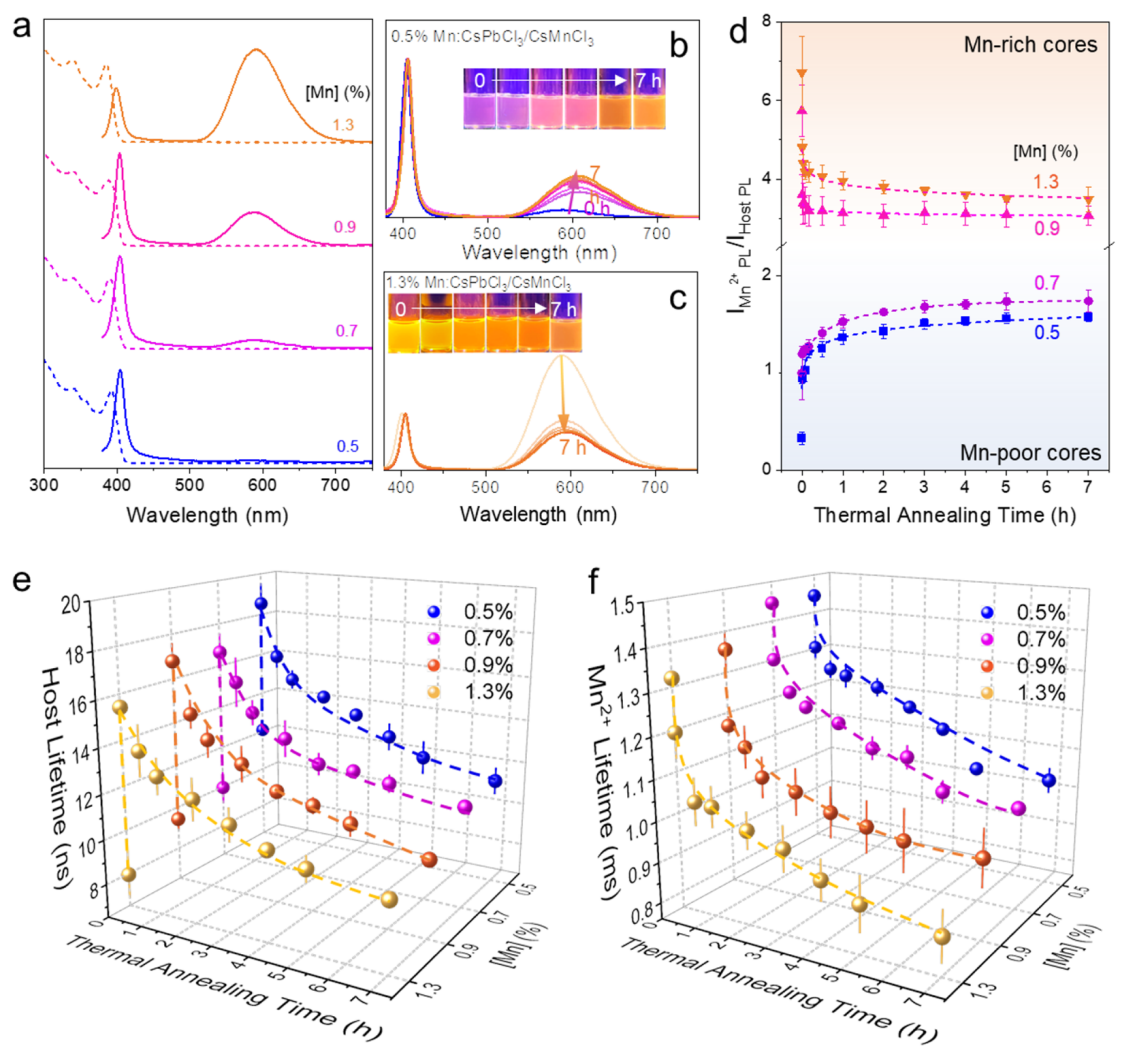
(a) PL spectra of Mn:CsPbCl_3_ NCs with 0.5, 0.7, 0.9, and 1.3% Mn doping concentration. PL spectra with different thermal annealing times (0–7 h) of (b) 0.5% Mn:CsPbCl_3_/CsMnCl_3_ core/shell NCs and (c) 1.3% Mn:CsPbCl_3_/CsMnCl_3_. Insets are the optical images of the NCs under UV illumination. (d) Ratio between Mn^2+^ PL and host PL in Mn:CsPbCl_3_/CsMnCl_3_ core/shell NCs as a function of the thermal annealing time. (e) Host PL lifetime decays (in nanoseconds) and (f) Mn^2+^ PL lifetime decays (in milliseconds) for Mn:CsPbCl_3_/CsMnCl_3_ core/shell NCs as a function of the thermal annealing time. All errors were calculated as the standard deviation of a population of three repeated samples.

Considering the ion-concentration-gradient-dependent ion diffusion, to further prove the B-site ion diffusion in the perovskite core/shell NCs, a series of Mn:CsPbCl_3_ NCs were synthesized to grow the corresponding Mn:CsPbCl_3_/CsMnCl_3_ core/shell NCs. The smaller [Mn] gradient between the core and shell lattice is expected compared to that of the undoped CsPbCl_3_/CsMnCl_3_ core/shell NCs. The concentration of Mn dopants was controlled by varying the ratio of MnCl_2_ and PbCl_2_ precursors (i.e., 50%, 100%, 150%, and 200% of MnCl_2_ to PbCl_2_), which results in Mn:CsPbCl_3_ NCs with 0.5, 0.7, 0.9, and 1.3% Mn dopants. The UV–visible absorption and PL spectra are shown in Figure 5a, and the EPR spectra are included in Figure S3. Similar to the PL QY change of CsPbCl_3_/CsMnCl_3_ core/shell NCs, the PL QYs of Mn:CsPbCl_3_/CsMnCl_3_ core/shell NCs are enhanced compared to that of Mn:CsPbCl_3_ cores as well (Figure S4), e.g., 15.2% for 1.3% Mn:CsPbCl_3_ NCs vs. 30.2% for 1.3% Mn:CsPbCl_3_/CsMnCl_3_ core/shell NCs. It is worth noting that the ratio of Mn PL and host PL in the Mn:CsPbCl_3_/CsMnCl_3_ core/shell NCs not only changes with respect to the thermal annealing time but also varies according to the original doping concentration. For example, with the PL intensity of host NCs normalized, the Mn PL increased as the thermal annealing time increased for the 0.5% Mn:CsPbCl_3_/CsMnCl_3_ core/shell NCs (Figure 5b), which is due to the continuous increase of Mn concentration in the core, while it is maintained at a relatively low level. However, such a trend is opposite for 1.3% Mn:CsPbCl_3_/CsMnCl_3_ core/shell NCs (Figure 5c) due to the Mn–Mn short-range interactions and concentration quenching effects with high Mn^2+^ concentration in the core of the core/shell NCs. Chromaticity coordinates for 0.5, 0.7, 0.9, and 1.3% Mn:CsPbCl_3_/CsMnCl_3_ core/shell NCs are summarized in Figures S5–S8. The plots of the ratio between Mn^2+^ PL and host PL with respect to thermal annealing time for Mn lightly doped (i.e., 0.5% and 0.7% [Mn]) Mn:CsPbCl_3_/CsMnCl_3_ core/shell NCs show the same trend as that of the undoped CsPbCl_3_/CsMnCl_3_, increasing from 0.3 to 1.5 and from 0.9 to 2.1 for 0.5% and 0.7% Mn:CsPbCl_3_/CsMnCl_3_ core/shell NCs (Figure 5d, blue background), respectively. However, for the core/shell NCs with relatively Mn-richer cores (0.9% and 1.3% Mn:CsPbCl_3_/CsMnCl_3_ core/shell NCs), the ratio between the Mn^2+^ PL and host PL gradually decrease with respect to the thermal annealing time (Figure 5d, orange background).

Plots of average host PL lifetime and average Mn^2+^ PL lifetime with respect to the thermal annealing time are summarized in Figure 5e,f (see raw PL lifetime decays in Figures S9 and S10). It was found that the higher the Mn^2+^ doping concentration in the core, the shorter the detected host PL lifetime. For example, 0.5% Mn:CsPbCl_3_ has a host PL lifetime of 12 ns, while the lifetime is reduced to 8 ns in 1.3% Mn:CsPbCl_3_. The Mn^2+^ PL lifetime data show a shorter lifetime with a higher amount of Mn concentration (e.g., 1.45 and 1.31 ms for 0.5% Mn:CsPbCl_3_ and 1.3% Mn:CsPbCl_3_, respectively). Such a trend of reduced Mn PL lifetime remains at the same thermal annealing time for each core/shell NC during the thermal annealing process. Comparable to the trend of host PL lifetime change of undoped CsPbCl_3_/CsMnCl_3_ core/shell NCs with respect to thermal annealing time, all the host PL lifetimes were first enhanced by the growth of the CsMnCl_3_ layer due to the enhanced PL QYs, followed by a gradual decrease from the concentration quenching effect as the Mn^2+^ ions diffuse to the core. The trend of Mn^2+^ PL lifetime with respect to the thermal annealing time of Mn:CsPbCl_3_/CsMnCl_3_ core/shell NCs is also consistent with that of undoped CsPbCl_3_/CsMnCl_3_ core/shell NCs, i.e., the more Mn^2+^ diffused into the core, the shorter the Mn^2+^ PL lifetime.

Since significant Mn inward ion diffusion can occur for all core/shell NCs with predoped Mn in the core, we hypothesize that other factors in addition to the concentration gradient dependence must play a role in the B-site ion diffusion in our system. To further elucidate the mechanism of the B-site ion diffusion in the perovskite core/shell NCs, we have simulated the final Mn concentration in the core due to the B-site ion diffusion as a function of Pd vacancy concentration in the core lattice at 120 °C, which is the same thermal annealing temperature used in our experiments. In the simulation, we have modeled the interface core/shell lattice in a cubic perovskite unit cell with 4 corner-sharing PbCl_6_ octahedra (left side of the scheme in Figure S11b) and 4 corner-sharing MnCl_6_ octahedra (right side of the scheme in Figure S11b). A theoretical simulation suggests that the presence of Pd vacancies in the B-site has a substantial influence on the equilibrium Mn^2+^ doping concentration in the core CsPbCl_3_ NCs. For example, 0.12–0.16% Pd vacancies in the core could lead to ∼1% Mn doping concentration in CsPbCl_3_ at a temperature of 120 °C by using the calibration curve (Figure S11a). Solid-state diffusion involving vacancies has been reported previously in a II–VI group NC vacancy-assisted migration mechanism.^47,48^ More interestingly, it was found that the concentration of Mn^2+^ ions in the core scales linearly with the number of vacancy sites (Figure S11b). Therefore, it is reasonable to believe that the B-site ion diffusion could be influenced by both ion concentration gradient and presence of B-site vacancy. The B-site vacancy concentration, especially at the core/shell interface where ion diffusion first takes place, might not be affected by the predoped Mn ions in the CsPbCl_3_ core; therefore, Mn ion diffusion from the CsMnCl_3_ shell to the CsPbCl_3_ core could occur based on the vacancy mechanism, which could explain the observed inward Mn ion diffusion in the core/shell NCs with predoped Mn ions in the CsPbCl_3_ core.

**Figure 6:**
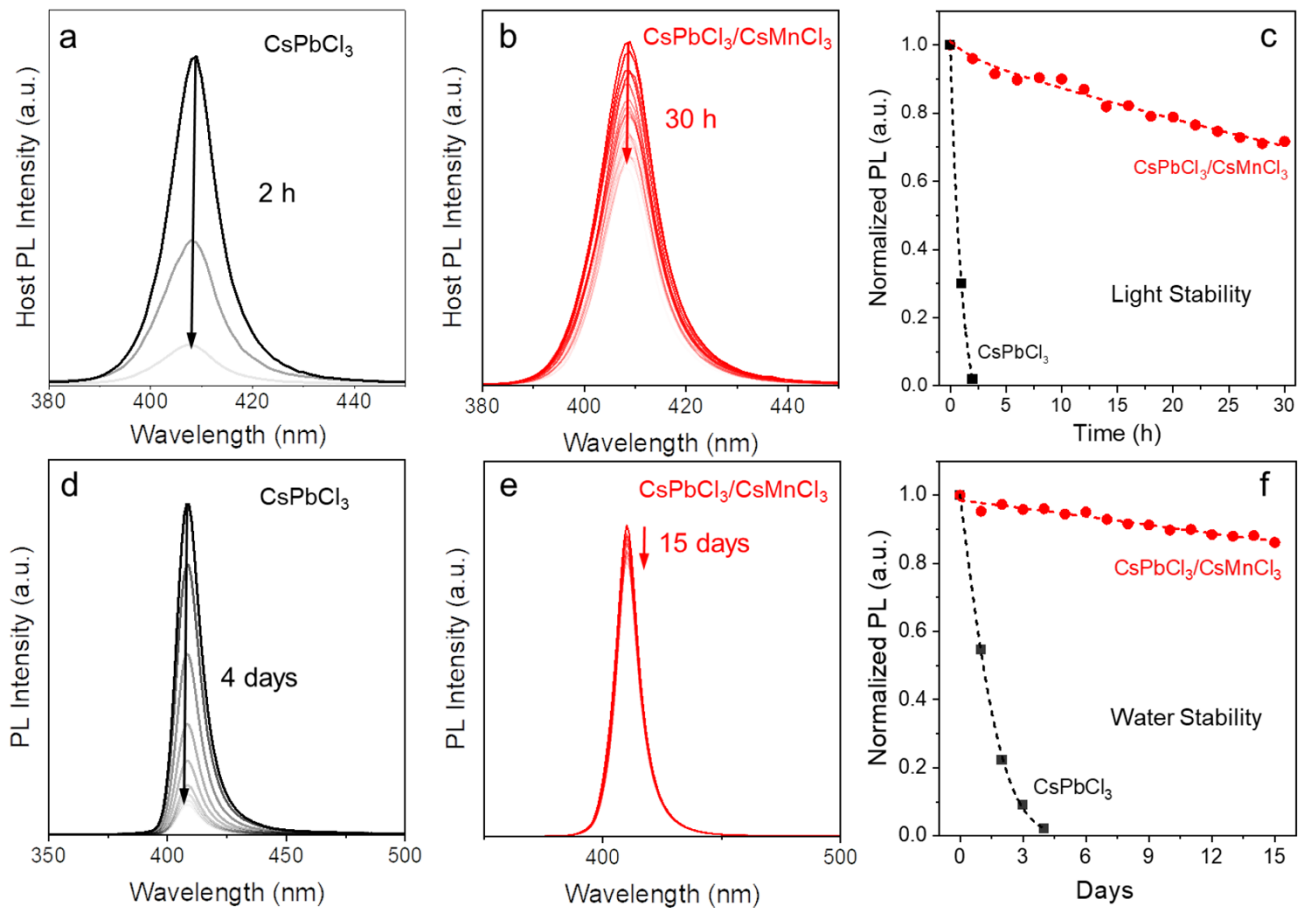
Photostability test of (a) CsPbCl_3_ NCs and (b) CsPbCl_3_/CsMnCl_3_ core/shell NCs monitored by host PL at 408 nm under UV light irradiation. (c) Normalized PL intensity of CsPbCl_3_ (black squares) and CsPbCl_3_/CsMnCl_3_ core–shell NCs (red dots) over UV irradiation time. Water stability tests of (d) CsPbCl_3_ NCs and (e) CsPbCl_3_/CsMnCl_3_ core/shell NCs. (f) Normalized PL intensity of CsPbCl_3_ (black squares) and CsPbCl_3_/CsMnCl_3_ core/shell NCs (red dots) over water treatment time monitored by host PL at 408 nm.

The designed lead-based/lead-free perovskite core/shell NCs not only have significant merits in tuning the optical properties but also can create environmentally friendly and stable perovskite-based materials. The photostability was tested for undoped CsPbCl_3_/CsMnCl_3_ core/shell NCs and 1.3% Mn:CsPbCl_3_/CsMnCl_3_ core/shell NCs (Figure 6a–c and Figure S12). While the CsPbCl_3_ NCs in toluene are very sensitive to UV light (365 nm, 10 ± 2 mW cm^–2^) and completely lose their PL intensity within 2 h (Figure 6a and black squares in Figure 6c), the PL intensity of the CsPbCl_3_/CsMnCl_3_ core/shell NCs in toluene gradually decrease and reach ∼72% after UV light irradiation for 30 h (Figure 5b and red dots in Figure 6c). For the 1.3% Mn:CsPbCl_3_ NCs and Mn:CsPbCl_3_/CsMnCl_3_ core/shell NCs, the PL intensity reaches almost 0 after UV illumination for 3 h for the former and can be maintained at ∼90% under UV light for 4 h for the core/shell NCs (Figure S12). This enhanced photostability of CsPbCl_3_/CsMnCl_3_ core/shell NCs is due to the excellent UV resistance of the CsMnCl_3_ shell^20^ and the type I structure of the core/shell NCs which protects the core NCs from photo-oxidation.^29,38^ The photostability was also tested using blue light with a stronger light optical power density, i.e., a 405 nm LED lamp with a light density of 250 ± 40 mW cm^–2^. The CsPbCl_3_ NCs in toluene are also sensitive to blue light and completely lose their PL intensity within 1.5 h (Figure S13a and black squares in Figure S13c). The CsPbCl_3_/CsMnCl_3_ core/shell NCs in toluene still show much enhanced light stability under blue light irradiation, as its PL intensity remains ∼80% after light irradiation using the 405 LED lamp for 4 h (Figure S13b and red dots in Figure S13c). Even though the higher light power can cause more photodegradation for the core and core/shell NCs, our photostability tests reveal that the CsPbCl_3_/CsMnCl_3_ core/shell NCs are great candidates for practical applications under different conditions, i.e., UV and blue-light irradiation.

The CsPbCl_3_/CsMnCl_3_ core/shell NCs also show excellent water stability compared to that of the unshelled CsPbCl_3_ NCs. CsPbCl_3_ NCs gradually lose the PL intensity during 4 days of water stability test (Figure 6d and black squares in Figure 6f), while CsPbCl_3_/CsMnCl_3_ core/shell NCs still retain ∼88% of the original PL intensity after 15 days in water (Figure 6e and red dots in Figure 6f). The improved water stability of the CsPbCl_3_/CsMnCl_3_ core/shell NCs is likely due to the great structural stability of CsMnCl_3_ under ambient conditions^49^ and the improved stability of the core NCs after Mn^2+^ dopant incorporation, as it was reported that doping into a perovskite lattice could significantly increase the structure stability of the host perovskite.^50^ The water stability of the CsMnCl_3_ shell materials was tested by the XRD measurement of the CsMnCl_3_ NCs after dispersion in water under continuous stirring (Figure S14). After 4 days of being submerged in water, the recovered CsMnCl_3_ crystals still preserved the main crystal structure (purple in Figure S14) as in the initial CsMnCl_3_ NCs (black in Figure S14). This incredible water stability could be ascribed to the formation of CsMnCl_3_(H_2_O)_2_ when suspending CsMnCl_3_ in water without destroying its main crystal structure,^51^ which could be recovered to anhydrous CsMnCl_3_ upon removing water by a general thermal treatment (∼80 °C in this study). Such an improvement of environmental stability for lead-based perovskite NCs via the passivation of a lead-free perovskite shell layer could contribute to the development of perovskite materials for color-tunable optoelectronic devices (e.g., LEDs) with long-term stability.

## Conclusion

This study fabricates lead-based CsPbCl_3_/lead-free CsMnCl_3_ perovskite core/shell NCs, which could effectively tune the photophysical properties and address the toxicity and poor stability of perovskites. Importantly, B-site ion (i.e., Pb^2+^ and Mn^2+^) exchange occurs at the interface of the core/shell NCs, rendering the formation of Mn-doped CsPbCl_3_ core NCs with increased Mn concentration from the ion exchange under thermal annealing. The formation of the Mn-doped CsPbCl_3_ core NCs after B-site ion exchange introduces a host-to-dopant energy transfer pathway in addition to excitonic recombination, which is a promising tool to tailor the photophysical properties of perovskite materials. The B-site ion diffusion/exchange in the CsPbCl_3_/CsMnCl_3_ core/shell NCs with a much lower toxicity and higher stability lead-free perovskite shell introduces desirable strategies for the development of perovskite-based functional materials for practical applications in more efficient and durable optoelectronic devices.

## Experimental Section

### Chemicals

Cesium carbonate (Cs_2_CO_3_, 99.995%,), oleic acid (OA, 90%), 1-octadecene (ODE, 90%), lead chloride (PbCl_2_, 99.999% trace metal), manganese chloride tetrahydrate (MnCl_2_·4H_2_O, 99.999%), oleylamine (OAm, 70%), trioctylphosphine (TOP, 90%,), toluene (99.6%), and methyl acetate (MA, 99%) were purchased and used without further purification.

### Preparation of Cesium Oleate Precursor

Cesium oleate stock solution (Cs-oleate) was prepared following a previously reported procedure.^33^ Briefly, Cs_2_CO_3_ (163 mg, 0.5 mmol) was mixed with OA (0.5 mL) and ODE (8 mL) in a 25 mL three-neck round-bottom flask. The mixture was dried in vacuo at 120 °C for 1 h, followed by thermal treatment under argon at 150 °C until Cs_2_CO_3_ and OA completely reacted for approximately 30 min. The Cs-oleate solution was then stored at room temperature and was preheated to ∼110 °C before further use.

### Synthesis of CsPbCl_3_ Core NCs

The CsPbCl_3_ NCs were synthesized following a previously reported procedure.^33^ Briefly, PbCl_2_ (104 mg, 0.376 mmol), OA (1 mL), OAm (1 mL), and ODE (10 mL) were loaded into a 25 mL three-neck round-bottom flask. 2 mL of TOP was added to solubilize PbCl_2_. The mixture was dried in vacuo at 120 °C for 1 h. After complete solubilization of PbCl_2_, the temperature was raised to 170 °C under argon, followed by swift injection of hot Cs-oleate solution (0.8 mL, 0.125 M in ODE, prepared as described above). Upon reaction for ∼5 s, the mixture was cooled to room temperature by using a water bath, and the CsPbCl_3_ NCs were collected by centrifuging at 500 rpm for 5 min. The crude NCs were then redissolved in a toluene/MA mixture with a volume ratio of 1:1 to wash out the unreacted salts and excess ligands. The purified NCs were then collected by centrifuging at 5000 rpm for 5 min and redissolved in toluene for further use.

### Synthesis of Mn-Doped CsPbCl_3_ Core NCs

The preparation of Mn:CsPbCl_3_ NCs with various Mn concentrations is similar to that of CsPbCl_3_ but using the salt mixture of PbCl_2_ and MnCl_2_·H_2_O.^7^ Briefly, PbCl_2_ (111 mg, 0.4 mmol), MnCl_2_·H_2_O (39 mg, 0.2 mmol; 78 mg, 0.4 mmol; 117 mg, 0.6 mmol, or 156 mg, 0.8 mmol), OA (3.2 mL), OAm (3.2 mL), TOP (2 mL), and ODE (10 mL) were loaded into a 50 mL three-neck round-bottom flask. The mixture was dried in vacuo at 120 °C for 1 h. After complete solubilization of PbCl_2_ and MnCl_2_, the temperature was raised to 170 °C under argon, followed by the swift injection of 0.8 mL of warm Cs-oleate solution preheated with a heat gun. Upon reaction for ∼5 s, the mixture was cooled to room temperature using a water bath, and the Mn:CsPbCl_3_ NCs were collected by centrifuging at 500 rpm for 5 min. The crude NCs were then redissolved in a toluene/MA mixture with a volume ratio of 1:1 to wash out the unreacted salts and excess ligands. The purified NCs were then collected by centrifuging at 5000 rpm for 5 min and redissolved in toluene for further use.

### Synthesis of CsMnCl_3_ NCs

The CsMnCl_3_ NCs were synthesized using a modified reported procedure.^20^ Briefly, MnCl_2_·4H_2_O (78 mg, 0.4 mmol), OA (1.5 mL), OAm (0.5 mL), and ODE (10 mL) were loaded into a 25 mL three-neck round-bottom flask and degassed in vacuo at 120 °C for 1 h. The solution was then heated to 140 °C under argon gas, and 0.8 mL of warm Cs-oleate precursor solution was swiftly injected into the reaction mixture. The reaction mixture was kept at 140 °C for ∼2 min until the solution mixture turned cloudy and then was cooled to room temperature using a water bath. The crude CsMnCl_3_ NCs were collected by centrifugation at 5000 rpm for 5 min, washed with a toluene/MA 1:1 mixture, and redissolved in toluene for further characterizations.

### Synthesis and Thermal Annealing of CsPbCl_3_/CsMnCl_3_ and Mn:CsPbCl_3_/CsMnCl_3_ Core/Shell NCs

In a typical synthesis, MnCl_2_ (39 mg, 0.2 mmol), OA (0.75 mL), OAm (0.25 mL), and ODE (5 mL) were loaded into a 25 mL three-neck round-bottom flask. The mixture was dried in vacuo at 100 °C for 1 h. Then the as-prepared CsPbCl_3_ or Mn:CsPbCl_3_ core NCs were dissolved into 1 mL of the ODE and injected into the reaction mixture under argon at 100 °C. The mixture was degassed for an additional 10 min and heated to 120 °C. A preheated Cs-oleate precursor solution (0.4 mL) was injected drop-wise into the reaction mixture. The reaction mixture was then cooled to room temperature using a water bath, and the as-synthesized core/shell NCs were precipitated out by centrifugation at 5000 rpm for 5 min. The unreacted precursors and ligands were removed by dissolving the crude product in toluene and precipitating the core/shell perovskite NCs by centrifugation at 5000 rpm for 5 min.

The purified core/shell NCs were then redissolved in ODE and heated to 120 °C for thermal annealing up to 7 h under argon. Upon various thermal annealing times (i.e., 0.5 min, 2 min, 5 min, 10 min, 30 min, 1 h, 2 h, 3 h, 5 h, and 7 h), the reaction mixture was cooled to room temperature using a water bath. The obtained CsPbCl_3_/CsMnCl_3_ core/shell NCs could be collected by centrifugation at 5000 rpm for 5 min and stored in toluene or washed with a toluene/MA mixture to obtain solid powders for further characterizations.

### Computational Details

The migration of the Mn^2+^ dopant from the shell to the core region was investigated computationally using both density functional theory (DFT) and classical Monte Carlo simulation to calculate the temperature-dependent distribution coefficient (*K*_D_) of the dopant in the core regions of the quantum dot (eq 2).
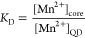
2The calculation of *K*_D_ was performed in three steps. In the first step, DFT calculations using the B3LYP and cc-pVDZ basis were performed using the TERACHEM computational package^55^ to calculate the relative energies of the site occupancy of the dopant in the octahedral site in the shell and core regions, respectively. These energy-minimization calculations helped to identify the most energetically favorable positions for the dopant. In the second step, additional DFT calculations were performed to sample the energies of other thermally accessible structures. These calculated energies allowed us to construct an effective electrostatic potential on a 3D real-space grid. In the third step, the generated effective potential was used to perform a canonical Monte Carlo calculation at *T* = 393 K to calculate the distribution coefficient. Assuming thermal equilibration, *K*_D_ was approximated as the relative population of finding the dopant in the core region of the QD (eq 3)
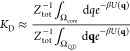
3where the total configuration integral (*Z*_tot_(*T*)) is defined over all space
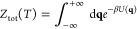
4The calculation of *K*_D_ was performed stochastically using the Metropolis–-Hastings algorithm where the trial move *q*_trial_ was accepted with the following probability:
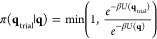
5The conditional probability mentioned above does not incorporate the influence of the vacancies within the core region. To include the presence of vacancies, a stochastic effective potential was introduced, and the potential within the core region was modified using the equation
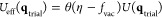
6where η∈[0,1] is a uniformly and identically distributed random variable and *f*_vac_ is the fraction of the vacancies in the core. The Monte Carlo simulation was performed for the range of vacancy fractions *f*_vac_∈[0.00, 3 × 10^–4^, 5 × 10^–4^, ..., 15 × 10^–4^] and a total of *N*_sample_ = 10^9^ sampling points were used for each value of *f*_vac_. To enhance accuracy, a segmented sampling approach was employed where the entire set of one billion sampling points was divided into 1000 segments, each containing one million independent sampling points. Sample mean and variance were calculated for each segment to estimate the total mean and variance of the calculated *K*_D_.

### Stability Tests

Photostability tests of CsPbCl_3_ NCs, Mn:CsPbCl_3_ NCs, and the corresponding core/shell NCs were performed by dissolving the perovskite NCs in toluene and exposing the NCs under 365 nm UV irradiation (10 ± 2 mW cm^–2^) and a blue LED (405 nm) with a much higher optical power density (250 ± 40 mW cm^–2^). Then, the PL intensity of the perovskite NCs was monitored at 408 nm over time. Water stability was tested by mixing the perovskite NCs solution in toluene with water (volume ratio = 1:1)^52,53^ under vigorous stirring to ensure sufficient interfacial interaction between water and perovskite NCs. Then the PL intensity of the NCs was monitored at 408 nm to evaluate the water resistance over time.

### Sample Characterization

The morphology and size distribution of NCs were analyzed by TEM using a JEM JEOL-2100F instrument with an accelerating voltage of 200 kV. Powder XRD patterns were taken on a Bruker D2 Phaser with a LYKXEYE 1D silicon strip detector using Cu Kα radiation (λ = 1.5406 Å). ICP-OES analysis was performed on a PerkinElmer Optama 3300 DV instruet. Room-temperature X-band EPR spectra were recorded at a microwave frequency of 9.4 GHz on a Bruker ELEXSYS-II E500 spectrometer. Absorption spectra were collected using an Agilent Cary 60 spectrophotometer. The PL measurements were conducted with a Horiba FluoroMax Plus spectrofluorometer. Time-resolved emission measurements were conducted using time-correlated single photon counting (TCSPC) for host PL and a mF2 60 W xenon flashlamp for Mn PL on an Edinburgh FLS-980 spectrophotometer. The lifetimes detected from time-resolved photoluminescence measurements were calculated using eq 7
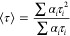
7where ⟨τ⟩ is experimentally detected by PL decay, α_*i*_ is the fractional amplitude of component *i*, τ_*i*_ is the lifetime of component *i*, and *i* is the number of exponentials.

## Supporting Information Available

All characterization data including TEM for shell perovskite NCs, EPR for the doped core NCs, PL spectra and chromaticity coordinates for the core/shell NCs, PL QYs, and stability tests of the doped core/shell NCs (PDF)
