# Supplementary material for: Interfacial B-Site Ion Diffusion in All-Inorganic Core/Shell Perovskite Nanocrystals
Source: ACS Nano. 2023 Nov 14;17(22):22467–77. doi: 10.1021/acsnano.3c05876 (PMC10690799; doi:10.1021/acsnano.3c05876)
Supplement: Supplementary file 1 — nn3c05876_si_001.pdf [file nn3c05876_si_001.pdf]

Supporting Information for

Interfacial B-Site Ion Diffusion in All-Inorganic Core/Shell Perovskite Nanocrystals

*Shuya Li,[a] Hanjie Lin,[a] Chun Chu,[a] Chandler Martin,[b] Walker MacSwain,[a] Robert W. Meulenberg,[c] John M. Franck,[a] Arindam Chakraborty,[a] Weiwei Zheng[a]**

[a]Department of Chemistry, Syracuse University, Syracuse, New York 13244, United States

[b]Department of Physics, Syracuse University, Syracuse, New York 13244, United States

[c]Department of Physics and Astronomy and Frontier Institute for Research in Sensor Technologies, University of Maine, Orono, Maine 04469, United States

Table of Contents

A. Morphology of $CsMnCl_3$ NCs S3

Figure S1. TEM images of $CsMnCl_3$ NCs. S3

B. PL QYs during Thermal Annealing S4

Figure S2. PL QYs of $CsPbCl_3$/$CsMnCl_3$ core/shell NCs with respect to the thermal annealing time .. S4

C. Electron Paramagnetic Resonance (EPR) Analysis S5

Figure S3. EPR spectra of Mn:CsPbCl3 core NCs with different doping concentrations S5

D. $CsMnCl_3$ Shell Passivation of Mn-Doped $CsPbCl_3$ NCs S6

Figure S4. PL QYs of core and core/shell NCs S6

Figure S5. PL and chromaticity coordinates of 0.5% Mn:$CsPbCl_3$/$CsMnCl_3$ core/shell NCs S7

Figure S6. PL and chromaticity coordinates of 0.7% Mn:$CsPbCl_3$/$CsMnCl_3$ core/shell NC S7

Figure S7. PL and chromaticity coordinates of 0.9% Mn:$CsPbCl_3$/$CsMnCl_3$ core/shell NCs S8

Figure S8. PL and chromaticity coordinates of 1.3% Mn:$CsPbCl_3$/$CsMnCl_3$ core/shell NCs S8

Figure S9. Host PL lifetime decays for Mn:$CsPbCl_3$/$CsMnCl_3$ core/shell NCs S9

Figure S10. Mn PL lifetime decays for Mn:$CsPbCl_3$/$CsMnCl_3$ core/shell NCs S10

E.Simulation Studies S11

Figure S11. Simulation of vacancy-assisted ion diffusion in the $CsPbCl_3$/$CsMnCl_3$ core/shell NCs .. S11

G. Stabilities Tests S12

Figure S12. Photostability (UV illumination) tests of Mn:$CsPbCl_3$ and Mn:$CsPbCl_3$/$CsMnCl_3$ core/shell NCs S12

Figure S13. Photostability (405 nm LED illumination) tests of $CsPbCl_3$ and $CsPbCl_3$/$CsMnCl_3$ core/shell NCs S12

Figure S14. Water resistance test of the $CsMnCl_3$ shell materials S13

A. TEM and HRTEM Images

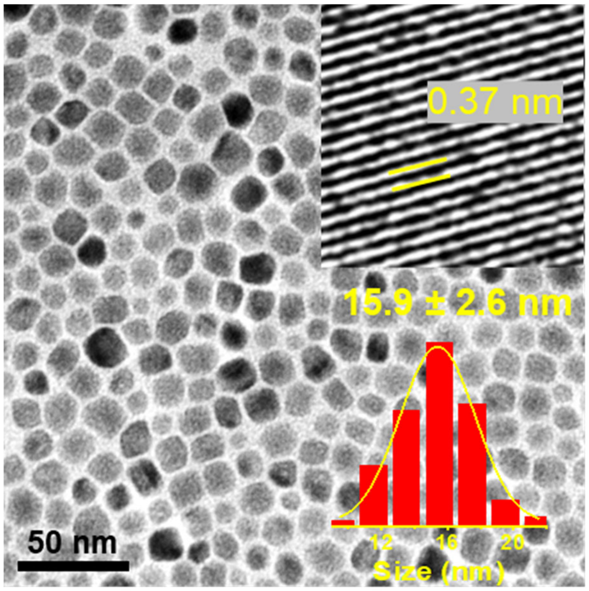

Figure S1. TEM image of $CsMnCl_3$ NCs, insets show the histogram of particle size and a high-resolution image shows *d*-spacing value of 0.37 nm for the cubic (110) plane (overlapped with orthorhombic (020) plane) of $CsMnCl_3$.

B. PL QYs during Thermal Annealing

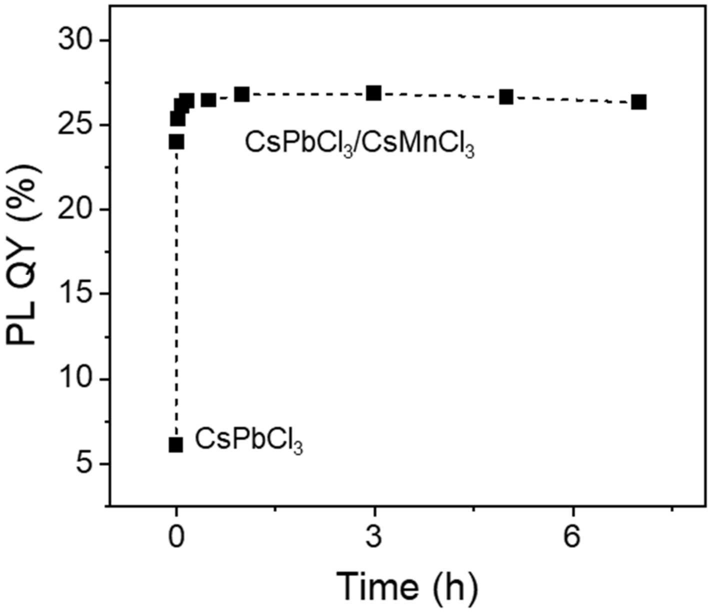

Figure S2. PL QYs of $CsPbCl_3/CsMnCl_3$ core/shell NCs with respect to the thermal annealing time.

C. Electron Paramagnetic Resonance (EPR) Analysis

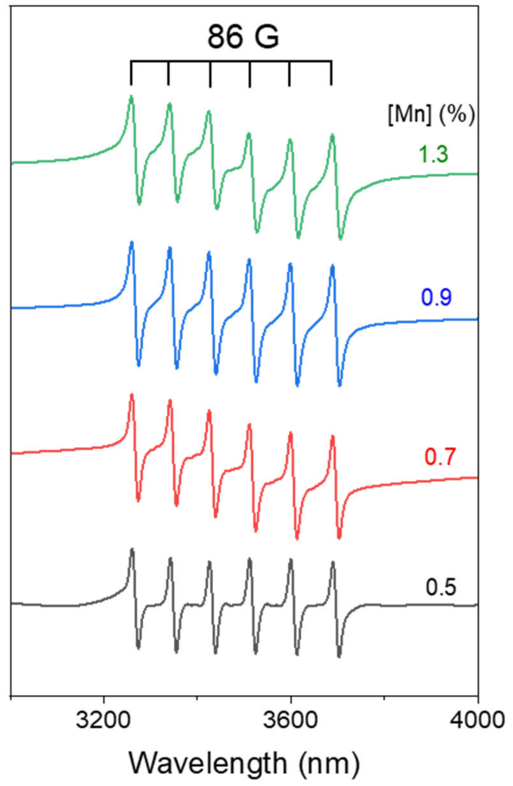

Figure S3. Room temperature X-band EPR spectra of Mn:$CsPbCl_3$ NCs with different doping concentrations ([Mn]: 0.5, 0.7, 0.9, and 1.3%).

D. $CsMnCl_3$ Shell Passivation of Mn-Doped $CsPbCl_3$ NCs

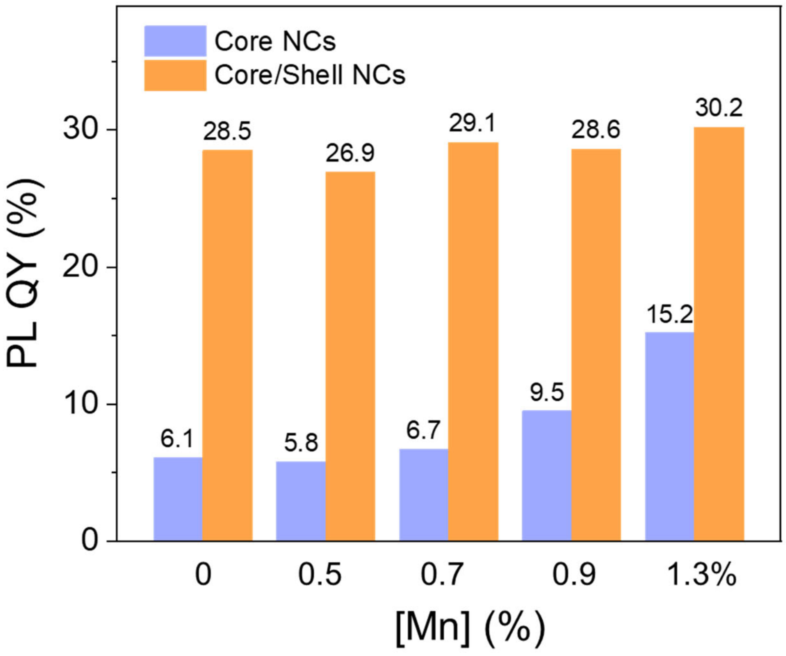

Figure S4. PL QYs of $CsPbCl_3$ NCs, $CsPbCl_3/CsMnCl_3$ core/shell NCs, Mn:$CsPbCl_3$ NCs and Mn:$CsPbCl_3/CsMnCl_3$ core/shell NCs with different Mn doping concentrations (0.5, 0.7, 0.9, and 1.3%).

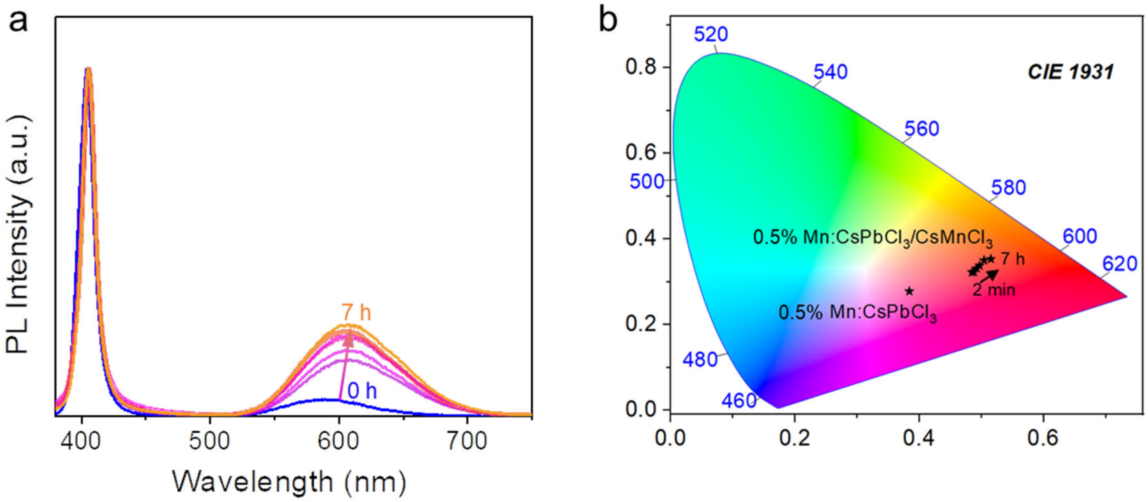

Figure S5. (a) PL spectra and (b) chromaticity coordinates of 0.5% Mn:$CsPbCl_3$ NCs and corresponding Mn:$CsPbCl_3$/$CsMnCl_3$ core/shell NCs with different thermal annealing times (0 – 7 h).

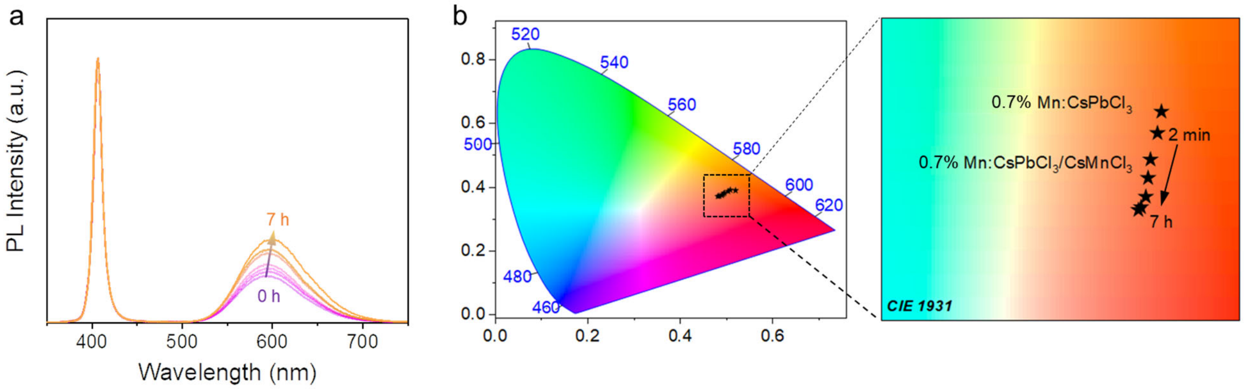

Figure S6. (a) PL spectra and (b) chromaticity coordinates of 0.7% Mn:$CsPbCl_3$ NCs and corresponding Mn:$CsPbCl_3$/$CsMnCl_3$ core/shell NCs with different thermal annealing times (0 – 7 h).

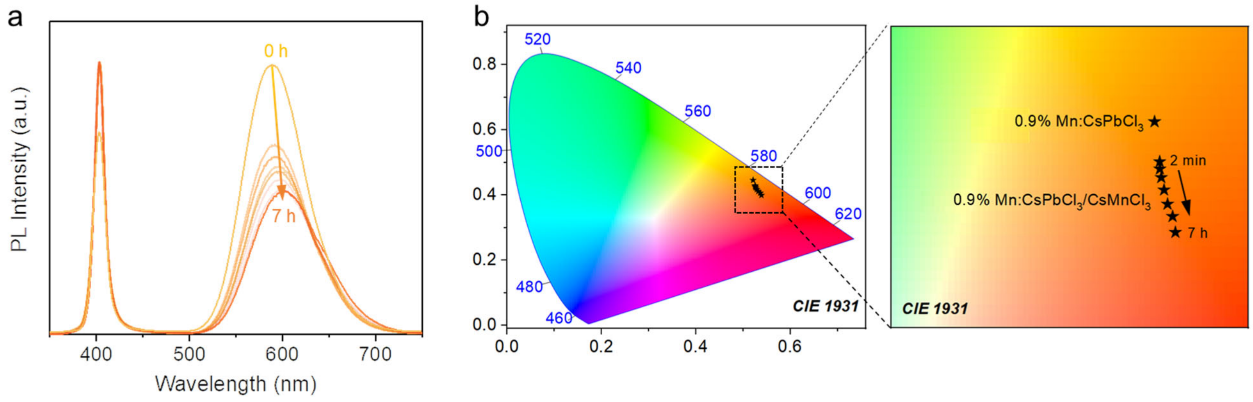

Figure S7. a) PL spectra and (b) chromaticity coordinates of 0.9% Mn:$CsPbCl_3$ NCs and corresponding 0.9% Mn:$CsPbCl_3$/$CsMnCl_3$ core/shell NCs with different thermal annealing times (0 – 7 h).

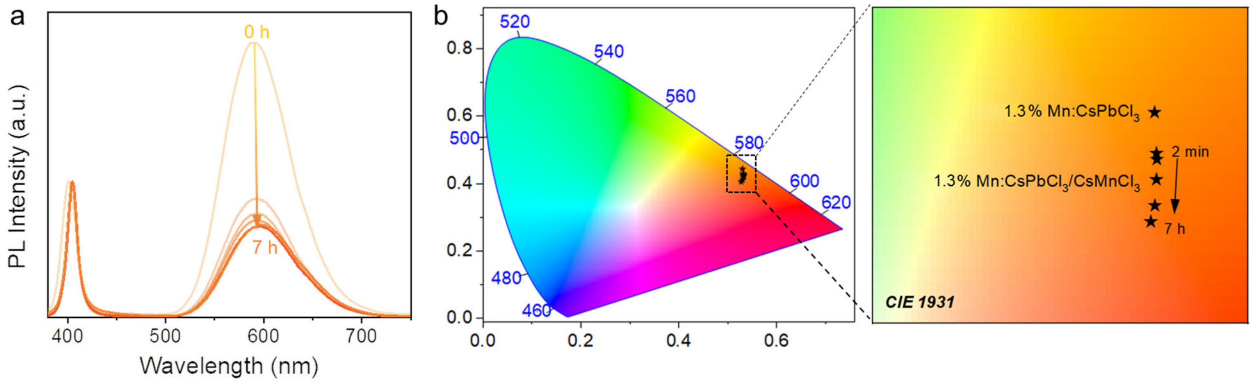

Figure S8. (a) PL spectra and (b) chromaticity coordinates of 1.3% Mn:$CsPbCl_3$ NCs and corresponding Mn:$CsPbCl_3$/$CsMnCl_3$ core/shell NCs with different thermal annealing times (0 – 7 h).

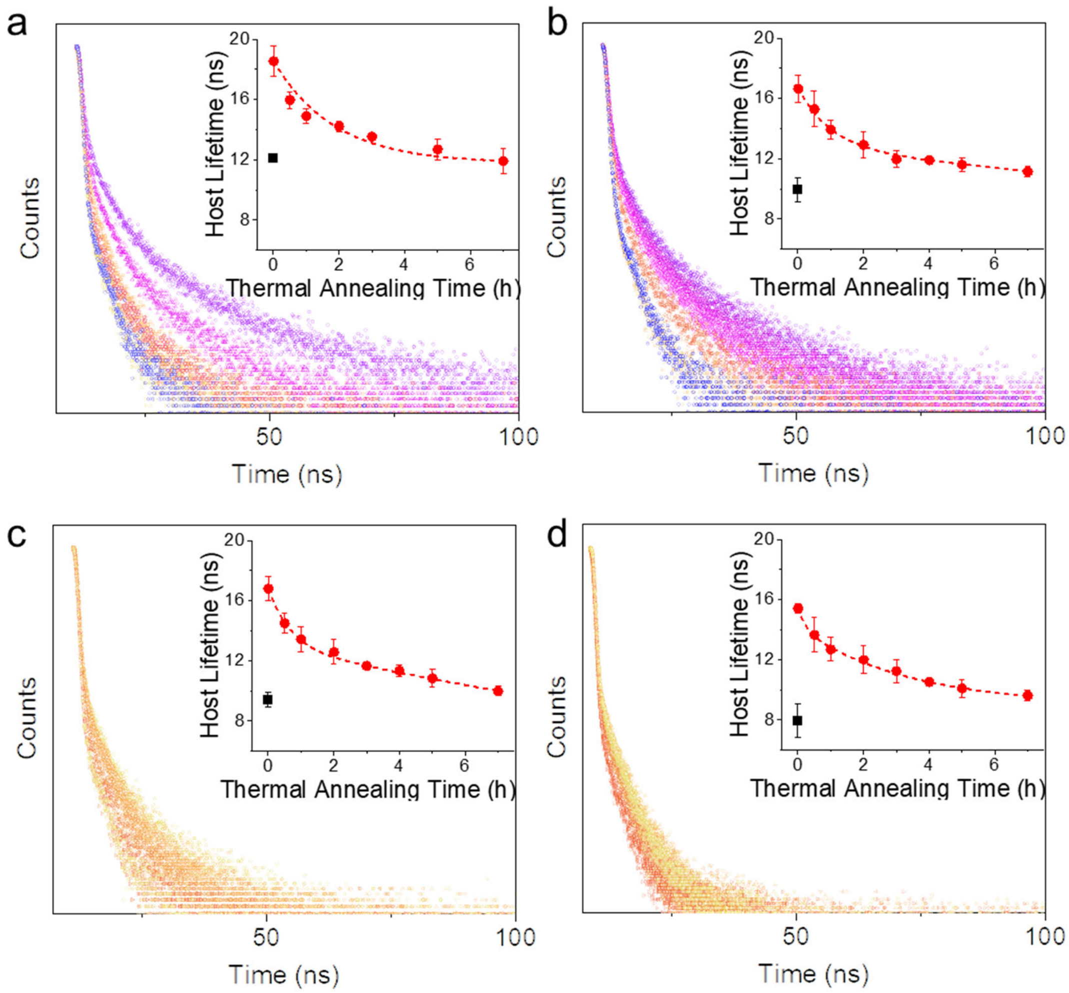

Figure S9. Host PL lifetime decays for Mn:$CsPbCl_3$/$CsMnCl_3$ core/shell NCs with different Mn doping concentration, (a) 0.5%, (b) 0.7%, (c) 0.9%, and (d) 1.3%, with respect to thermal annealing time. All errors were calculated as the standard deviation of a population of three repeated samples.

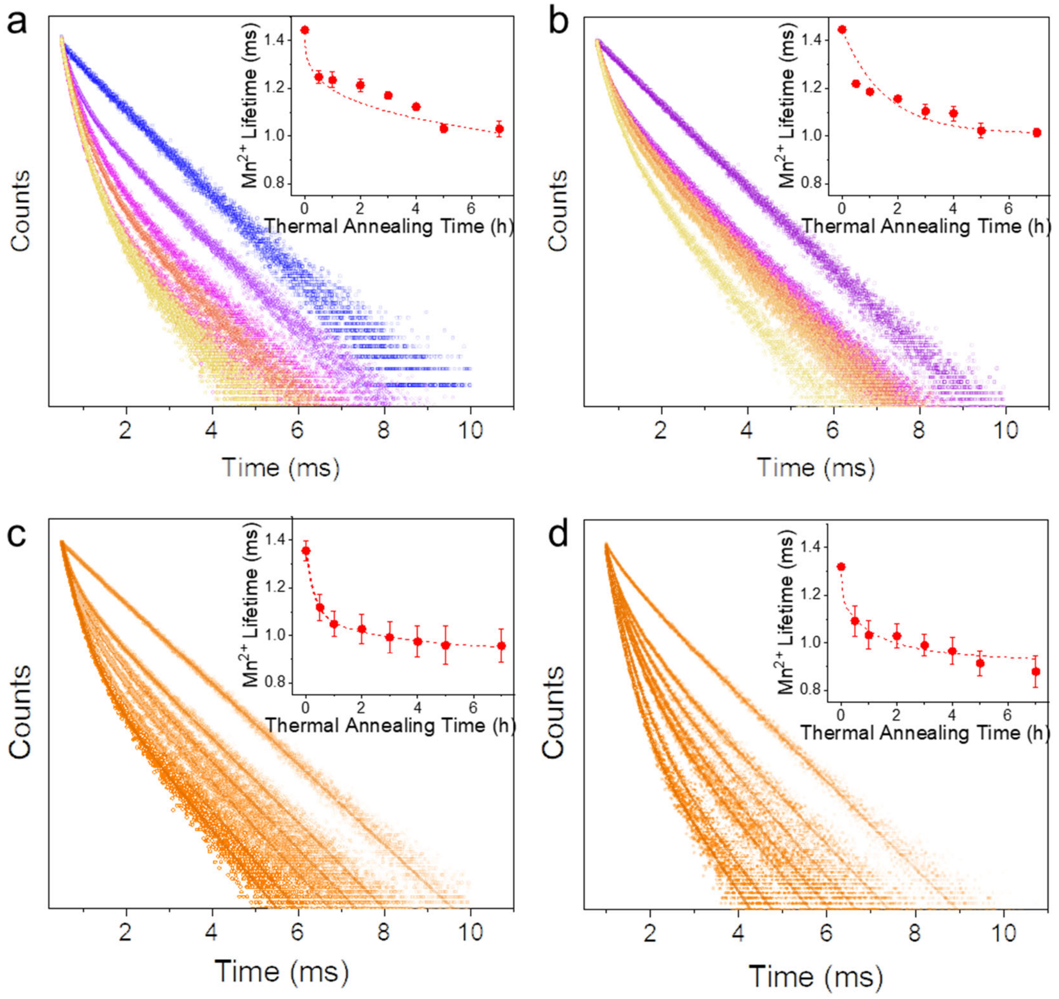

Figure S10. Mn PL lifetime decays for Mn:$CsPbCl_3$/$CsMnCl_3$ core/shell NCs with different Mn doping concentration, (a) 0.5%, (b) 0.7%, (c) 0.9%, and (d) 1.3%, with respect to thermal annealing time. All errors were calculated as the standard deviation of a population of three repeated samples.

E. Simulation Studies

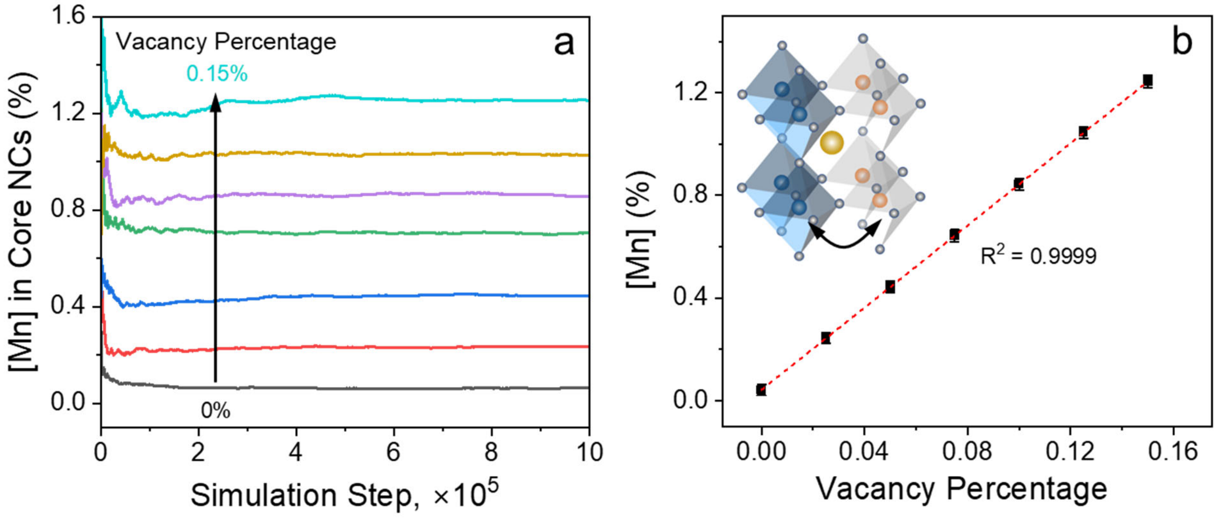

Figure S11. (a) Mn^{2+} doping concentrations in the $CsPbCl_3$ core NCs with various vacancy percentage in the core NCs at the core/shell interface over simulation step. (b) The equilibrium of Mn^{2+} concentration in the $CsPbCl_3$ core NCs with respect to the vacancy percentage. Inset: the simulated model lattice in a cubic perovskite unit cell with 4 corner-sharing $PbCl_6$ octahedra (left side) and 4 corner-sharing $MnCl_6$ octahedra (right side).

F. Stabilities Tests

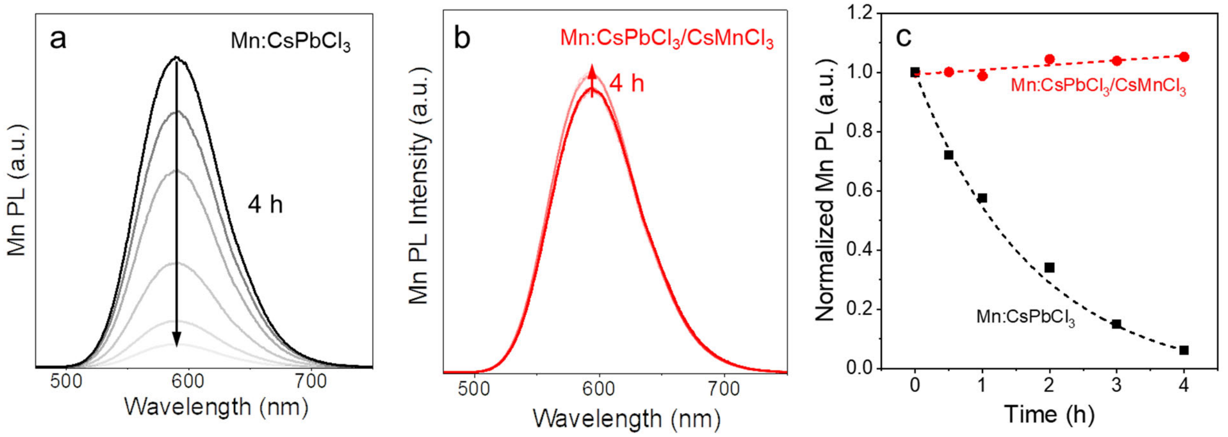

Figure S12. PL intensity over UV light irradiation time for photostability test of (a) 1.3% Mn:CsPbCl3 NCs and (b) 1.3% Mn:CsPbCl3/CsMnCl3 core/shell NCs monitored by Mn^{2+} emission at ~600 nm. (c) Normalized PL intensity of 1.3% Mn:CsPbCl3 (black squares) and 1.3% Mn:CsPbCl3/CsMnCl3 core shell NCs (red dots) over light irradiation time. All samples were stored and measured PL emissions in toluene.

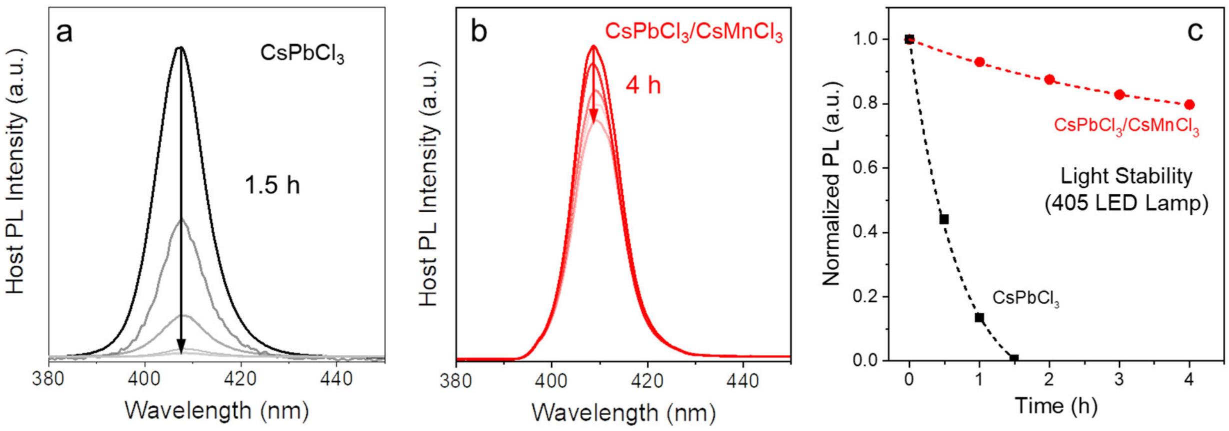

Figure S13. Photostability test of (a) CsPbCl3 NCs and (b) CsPbCl3/CsMnCl3 core/shell NCs monitored by host PL at 408 nm under visible light irradiation (405 nm LED Lamp). (c) Normalized PL intensity of CsPbCl3 (black squares) and CsPbCl3/CsMnCl3 core/shell NCs (red dots) over light irradiation time.

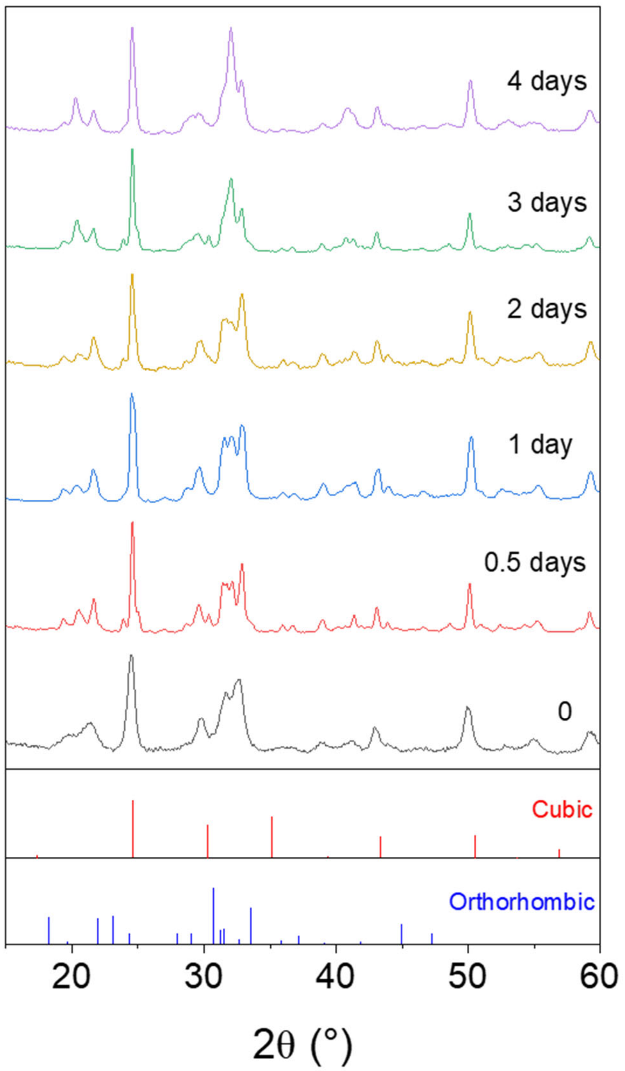

Figure S14. XRD patterns for the water resistance test of the $CsMnCl_3$ shell materials over 4 days. The reference XRD patterns of cubic and orthorhombic phases of $CsMnCl_3$ crystals were also included.
